# Plasmonic Polymorphs by Combining Shape Anisotropy and Soft Interactions in Bipyramid Thin Films

**DOI:** 10.1002/smll.202500389

**Published:** 2025-05-30

**Authors:** Jules Marcone, Sabrina Juergensen, Juan Barrios‐Capuchino, Xiaoyian Li, Claire Goldmann, Andrea Köppen, Winnie Pfeiffer, Felix Lehmkühler, Wolfgang J. Parak, Mathieu Kociak, Marianne Impéror‐Clerc, Stephanie Reich, Cyrille Hamon, Florian Schulz

**Affiliations:** ^1^ Laboratoire de Physique des Solides Université Paris‐Saclay CNRS Orsay 91405 France; ^2^ Department of Physics Freie Universität Berlin 14195 Berlin Germany; ^3^ Institute of Nanostructure and Solid State Physics Universität Hamburg 22761 Hamburg Germany; ^4^ Institute of Physical Chemistry Universität Hamburg 20146 Hamburg Germany; ^5^ Deutsches Elektronen‐Synchrotron DESY 22607 Hamburg Germany

**Keywords:** electron energy loss spectroscopy, high‐resolution transmission electron microscopy, moiré lattices, multilayers, self‐assembly

## Abstract

Thin‐film plasmonic supercrystals of pentagonal gold nanobipyramids (AuBP) exhibit a diverse range of packing structures that influence the near‐field distribution of the enhanced electric field and the far‐field response. By varying the molecular weight of the coating ligands, the softness of the anisotropic building blocks is changed. A thorough structural characterization reveals that this affects the resulting superstructures from self‐assembly more intricately than with isotropic building blocks. Softer coatings lead to smaller aligned domains in monolayers, while bilayers exhibit more crystalline domains with dominant interlayer twist angles near 0° and 90°. The far‐field distribution and near‐field response are measured using micro‐absorbance and electron energy loss spectroscopy (EELS). Correlating these data with high‐resolution transmission electron microscopy (HR‐TEM) structural analysis enabled the identification of the longitudinal and transverse individual and collective plasmonic modes. Notably, for large crystalline bilayer domains, a strong polarization‐dependent optical response is observed. These features underline the potential of these superstructures for applications in surface‐enhanced spectroscopies, plasmonic photocatalysis, and advanced optical manipulation in switchable optical metamaterials.

## Graphical abstract

The interplay of soft ligand coatings and anisotropy leads to structural diversity in superstructures of pentagonal gold nanobipyramids (AuBP). The correlated analysis of structure and optical properties with high‐resolution transmission electron microscopy (HR‐TEM), scanning transmission electron microscopy electron energy loss spectroscopy (STEM‐EELS), and micro‐absorbance spectroscopy reveals heterogeneous and anisotropic near‐field distributions and a strongly polarization‐dependent far‐field response.

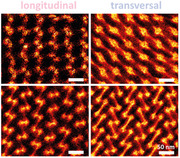

## 1 Introduction

Supercrystals of nanoparticles are fascinating artificial structures for tuning material properties and creating novel emergent properties.^[^ ^1^ ^]^ Plasmonic nanoparticle building blocks are particularly noteworthy for the manipulation of light, achieving deep strong light‐matter coupling in densely packed supercrystals and extreme focusing of the electric field component of light.^[^ ^2^ ^]^ A well‐established application of such light focused into nanoscale hot spots is advanced plasmon‐enhanced spectroscopies like surface‐enhanced Raman scattering (SERS).^[^ ^3^ ^]^ Another very promising direction is plasmonic photocatalysis where the optimized supercrystals may lead to minimal plasmonic losses and maximal photocatalytic activity.^[^ ^4^ ^]^ It has been shown that the locally enhanced electric fields can play a decisive role in plasmonic photocatalysis.

In addition to the lattice structure of the supercrystals and the material of the nanoparticle, nanoparticle shape, and coating offer additional parameters that contribute to the vast structural diversity that is being explored.^[^ ^5^ ^]^ In the case of anisotropic plasmonic nanoparticles, their shape anisotropy leads to a more complex optical response and accordingly, this structural diversity also translates to a diversity of plasmonic and optical properties. Switchable near‐field distributions, polarization‐dependent absorption, and facet‐dependent emission of incorporated dye molecules are notable examples.^[^ ^6^ ^]^ In this context, pentagonal gold nanobipyramids (AuBP) are an excellent candidate to study the interplay of shape anisotropy and ligand effects in the formation of complex plasmonic superlattices. They feature both, an interesting and well‐defined structure and assembly behavior, as well as distinct plasmonic properties.

AuBP feature strongly enhanced electric fields at the tips due to radius‐of‐curvature effects, resulting in intense hotspots.^[^ ^7^ ^]^ They can be produced with narrow size distributions, leading to narrow localized surface‐plasmon resonances (LSPR) due to reduced inhomogeneous broadening compared to, e.g., gold nanorods.^[^ ^8^ ^]^ The pentagonal cross‐section of AuBP varies along the longitudinal axis, which typically leads to low symmetry arrangements in closed‐packed lattices.^[^ ^9^ ^]^ Among the various methods that have been employed to induce the formation of NP superstructures, evaporation‐induced self‐assembly at an air/liquid interface provides a genuine way toward extended superlattices for different nanostructure building blocks, facilitates their transfer onto arbitrary substrates, and allows for the preparation of freestanding films.^[^ ^10^ ^]^ This method often produces moiré patterns in bi‐ and trilayers that are intriguing for the strong dependence of their physical properties on the twist angle between the layers.^[^ ^11^ ^]^ For NPs with circular cross‐section, control over twist angles is challenging to achieve and the layers are randomly stacked on top of each other.^[^ ^11^, ^12^ ^]^ On the other hand, polymorphous packing was observed for other NPs such as nanodumbbells,^[^ ^13^ ^]^ nanoarrows,^[^ ^14^ ^]^ tetrahedra,^[^ ^15^ ^]^ beveled triangles^[^ ^16^ ^]^ and pentagonal nanoprims.^[^ ^17^ ^]^ Although AuBP were assembled into various structures in the past,^[^ ^6^, ^18^ ^]^ their long‐range assemblies at the air/liquid interface into polymorphous moiré lattices has not been realized. Correlated structural and optical analyses have been explored in simpler isotropic systems, e.g. through the combination of transmission electron microscopy (TEM) and electron energy loss spectroscopy (EELS), but their application to anisotropic multilayer superstructures with collective plasmon modes remains rare.^[^ ^19^ ^]^ Here, we build on previous studies of 3D and monolayer assemblies by investigating the structure and optical properties of self‐assembled AuBP thin films, uncovering a range of novel configurations, particularly in bilayer systems.

We studied the formation of thin‐film superlattices of pentagonal AuBP at the air‐liquid interface. We observed different local structures in mono‐, bi‐, and multilayers, although with smaller grain sizes compared to isotropic gold nanoparticles (AuNP). Moiré lattices formed in bilayers with preferred twist angles *Θ* of 0° and 90° between AuBP layers. Reducing the coating thickness via the molecular weight of the polymer ligands resulted in larger monolayer domains with increased correlation of atomic lattice (AL) and superlattice (SL), but multilayers showed reduced order. The structure of the SLs was studied with TEM, high‐resolution TEM (HR‐TEM and their plasmonic properties with micro‐absorbance and EELS. The activation of collective plasmonic modes, their energy, and the relative intensity of longitudinal versus transverse excitations are all affected by the arrangement of the anisotropic building blocks. Our findings demonstrate how combining shape anisotropy and soft interactions may control the formation of lattice polymorphs with locked orientation. The lattice structures lead to interesting near‐field distributions like intense hot spots in voids of open lattices, which could inspire the design of plasmonic metasurfaces. Due to the anisotropy of the building blocks, both, near‐field distribution and far‐field optical response of ordered domains are polarization‐dependent and thus these plasmonic superstructures are excellent candidates for switchable plasmonic materials. The experimental work presented herein demonstrates the feasibility of obtaining complex superstructures based on anisotropic building blocks by straightforward self‐assembly. Furthermore, by providing a correlated near‐and far‐field optical characterization of these structures, this study offers new insights into the plasmonic behavior of complex nanoscale architectures, paving the way for future advancements in plasmonic material design.

## 2 Results and Discussion

### 2.1 AuBP Building Blocks and Self‐Assembly

**Figure 1:**
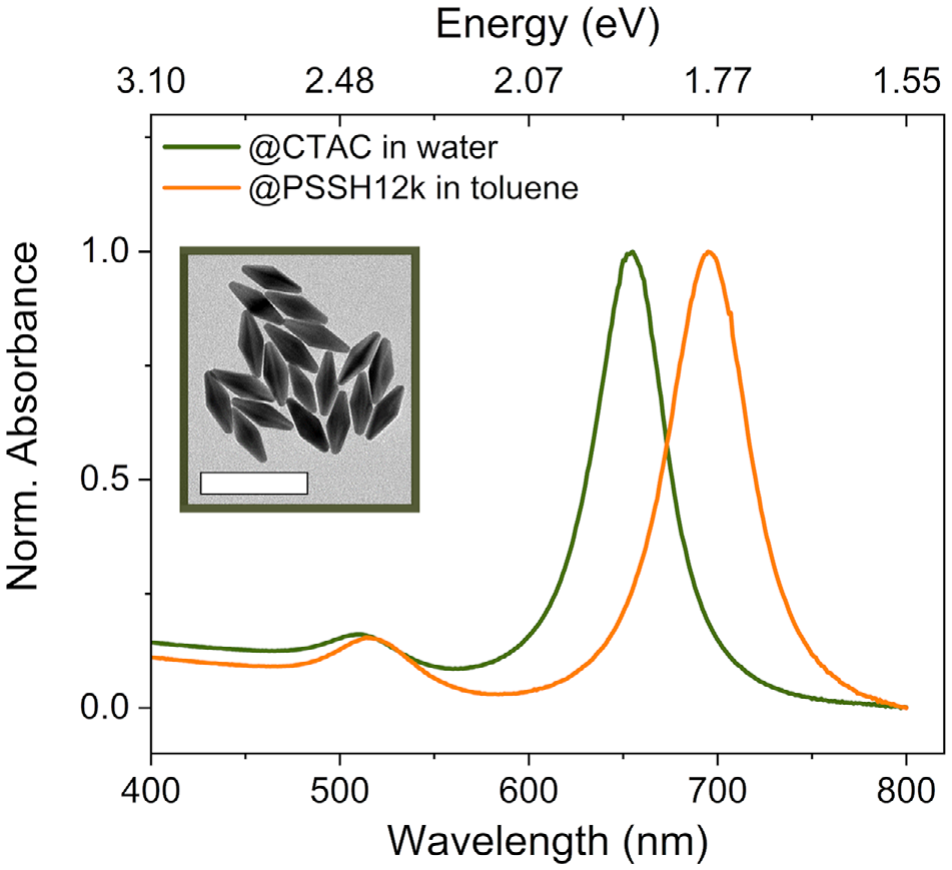
Normalized absorbance spectra of AuBP (long axis ≈60 nm, short axis ≈22 nm) stabilized with cetyltrimethylammonium chloride (CTAC, 10 mm) in aqueous dispersion (green) and the same BP coated with PSSH_12k_ in toluene (orange). The inset shows a TEM measurement of the starting material (AuBP@CTAC dried from dispersion in water), scale bar 100 nm.

The pentagonal AuBP as supercrystal building blocks are depicted in **Figure** **1** (inset) together with their optical characterization in aqueous dispersion and in the final purified dispersion in toluene.

The ligand exchange with thiol‐terminated polystyrene (PSSH_12k_, *M*_n_ ≈12 000 g mol^−1^, or PSSH_2k_, *M*_n_ ≈2000 g mol^−1^) was based on established protocols.^[^ ^11b^ ^]^ It yielded stable polystyrene‐coated bipyramids (AuBP@PSSH) that were phase‐transferred from water to toluene. The cetyltrimethylammonium chloride (CTAC) coated AuBP in aqueous dispersion (AuBP@CTAC) feature a distinct transverse and longitudinal plasmon resonance in the absorption spectra, which shifts to lower energies or longer wavelengths after transfer to toluene due to the change in refractive index. The transverse mode shifts from 512 nm in water to 516 nm in toluene (change of 20 meV in resonance energy), whereas the longitudinal mode shifts by more than 100 meV or from 654 to 695 nm. The refractive index sensitivity is related to the mode volume and local field enhancement; the stronger shift of the longitudinal mode is correlated with the strong enhancement at the tips of the AuBP. The plasmon resonance linewidth remains constant after transfer to toluene, which confirms that the AuBP remained well dispersed during ligand exchange. The spectra of AuBB@PSSH_2k_ in toluene were similar to those of AuBP@PSSH_12k_ (Figure S1, Supporting Information).

**Figure 2:**
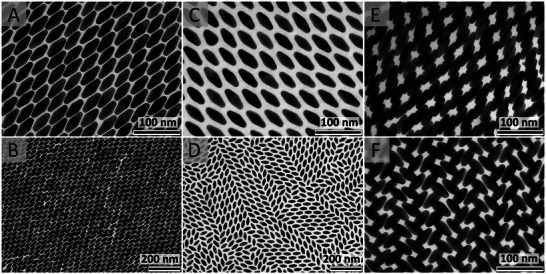
Different structures were observed in self‐assembled superlattices of AuBP@PSSH_2k_ (A and B) and AuBP@PSSH_12k_ (C‐F). A,B) Comparably large and densely packed aligned monolayers were observed for AuBP@PSSH_2k_ (scale bars A: 100 nm, B: 200 nm). C) Locally, the AuBP@PSSH_12k_ were also aligned in the monolayers (scale bar: 100 nm) but as shown in D), the domains were very small and mixed with amorphous domains (scale bar: 200 nm). The magnifications in A) and C) as well as B) and D) are identical allowing a comparison of the packing density. E,F) In bilayers of self‐assembled AuBP@PSSH_12k_ a variety of structures were observed with dominant twist angles of ≈0° and ≈90°. (scale bars 100 nm).

The self‐assembly of the AuBP@PSSH into supercrystals was induced on a liquid subphase by the slow evaporation of the solvent (toluene) on diethylene glycol,^[^ ^10^, ^20^ ^]^ resulting in a rich selection of superstructures, **Figure** **2**.

All samples showed well‐defined layered structures, similar to supercrystals of spherical AuNP produced by the same method.^[^ ^11b^ ^]^ In mono‐, bi‐ and trilayers of AuBP@PSSH_12k_ we observed amorphous domains with limited local order (Figures S2 and S3, Supporting Information), small aligned domains in monolayers (≈1 µm^2^ or smaller, Figure 2C,D), and ordered domains with varying crystal structure in bilayers (Figure 2E,F) and domain sizes up to tens of square micrometers (Figures S4–S9, Supporting Information). Considering the statistical limitations of TEM, quantitative interpretations are very hard, but bilayers tended to have larger domains with better long‐range order. The intriguing variety of local structures included densely packed and open domains and also different angles of alignment that clustered around 0° and 90° twist (Figure 2E,F; Figures S10 and S11, Supporting Information).

The structural variety of the AuPB supercrystals originates from the soft PSSH_12k_‐ligands; CTAC‐coated AuBP assemble into a well‐defined triclinic crystal structure that is rationalized by a maximization of the packing density.^[^ ^9^ ^]^ In complementary experiments, we assembled AuBP functionalized with the shorter PSSH_2k_ ligand (Figure 2A,B; Figures S12 and S13, Supporting Information). In these superlattices, the packing was denser, as expected, and the alignment in monolayers was much more pronounced. On the other hand, no local structures like those observed for AuBP@PSSH_12k_ were observed in bi‐ and multilayers. This observation supports the idea that a softer coating (here PSSH_12k_ being “softer” than PSSH_2k_‐based coatings) allows for more open lattices and structural diversity. When using a PSSH ligand with an intermediate molecular weight (PSSH_5k_, *M*_n_ ≈5000 g mol^−1^), neither pronounced alignment in monolayers as for AuBP@PSSH_2k_ nor large crystalline domains in bilayers as for AuBP@PSSH_12k_ were observed (Figure S14, Supporting Information). The reduced alignment in monolayers is in line with expectations (PSSH_5k_ being “softer” than PSSH_2k_‐based coatings) and the intermediate softness seems to prevent the formation of more open crystalline structures, possibly due to the insufficient deformability of the PSSH_5k_ coating preventing the formation of continuous films along with such open structures. Using ellipsoidal AuNP instead of AuBP resulted in less dense packing when coated with PSSH_12k_ compared to PSSH_2k_ as expected (Figure S15, Supporting Information). Some alignment and localized order were observed, but less pronounced than with the AuBP samples. We emphasize once more that interpretations and generalizations should be drawn with care, considering statistical limitations and the complexity of the parameter space. For example, it cannot be excluded that crystalline domains in bilayers or large aligned domains in monolayers can form with PSSH_5k_ even though they were not observed in our experiments. The complementary experiments underline that both: shape and coating play a role in structure formation in a delicate interplay.

### 2.2 Correlation of Atomic Lattices and Superlattices

An interesting aspect of supercrystals of nonspherical nanoparticles with well‐defined atomic crystal structures is the correlation of their AL with the resulting SL.^[^ ^1^, ^21^ ^]^ This correlation explains the role of the coating ligands during superstructure formation: For short ligands with comparably low molecular weights – alkyl ligands or cetyltrimethylammonium halides are popular choices – the crystal facets contribute strongly and even dominate particle‐particle interactions. This favors structures with maximum compacity, indicating an inclination toward entropy‐driven configurations.^[^ ^5^, ^20^, ^22^ ^]^ Soft coatings with polymers of large molecular weights, in contrast, lead to dominant ligand interaction between particles and less dependence on crystal facets. A mixed situation arises if the structure of the ligand coating is strongly affected by crystal facets, e.g., when the grafting density varies for different facets. This can be used to engineer the directional assembly of anisotropic nanoparticles, an example is chains of nanorods.^[^ ^2^, ^23^ ^]^

**Figure 3:**
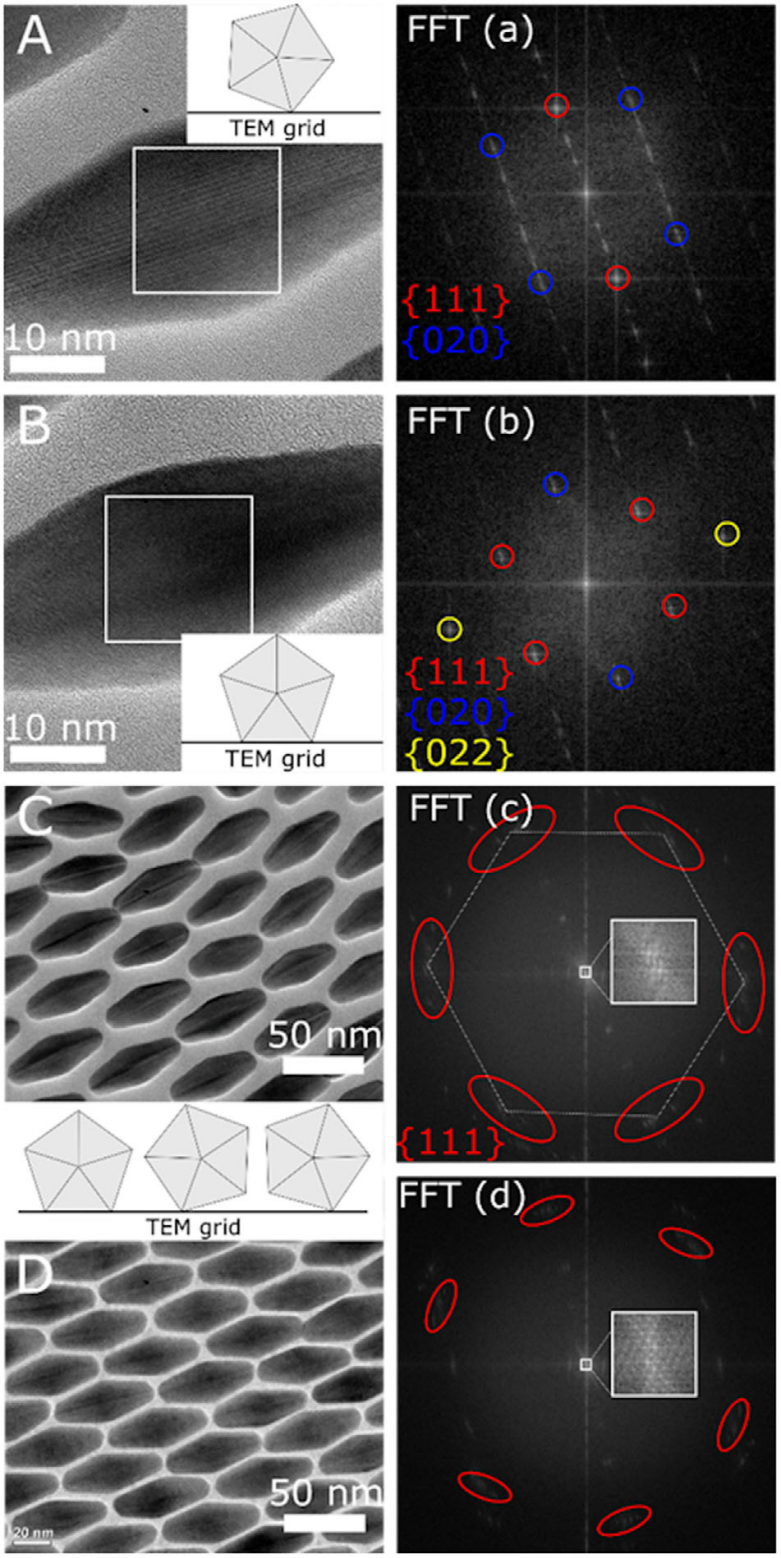
HR‐TEM characterization of the plasmonic superlattices. A–D) HR‐TEM measurements of the superlattice and corresponding FFT for A‐C) AuBP@PSSH_12k_ and D) AuBP@PSSH_2k_. From this analysis, the orientation of the bipyramids on the TEM grid is deduced and indicated in the schemes. For A) and B) the FFT was performed in the regions indicated by the white squares and for the whole image in C) and D). Inset in the FFT C) and D) shows the distribution of the spots corresponding to the nanostructures.

The azimuthal orientation of the AuBP in the thin films is observed in TEM. We determined the radial orientation of the bipyramids from the HR‐TEM measurements to identify a potential correlation between the AL and the SL (**Figure** **3**). The atomic crystal structure of the bipyramids features two fivefold pyramid bases stacked together, each composed of five single‐crystalline fcc domains. As a result, in the HR‐TEM measurements fringes appear, depending on the relative orientation of the bipyramids, Figure 3. As discussed by Johnson et al.^[^ ^24^ ^]^ and others^[^ ^25^ ^]^ this can be used to deduce the radial orientation of NPs with five‐fold symmetry on the substrate. We find that despite the long‐range orientation in the AuBP@PSSH superlattice, the bipyramid cross‐sections are randomly oriented, Figure 3. Again, this is in contrast to the finding on the AuBP@CTAC superlattices and attributed to the longer PSSH ligands.

AuBP@PSSH_2k_ represents an intermediate softness between the PSSH_12k_‐ and CTAC coatings because it is a much shorter ligand than PSSH_12k_. The thickness of the coatings in dispersion is ≈3.0 nm for CTAC,^[^ ^26^ ^]^ ≈3–4 nm for PSSH_2k_, and ≈8–9 nm^[^ ^11c^ ^]^ for PSSH_12k_. The values for the PSSH‐ligands were determined on spherical AuNP with dynamic light scattering which is not possible with the anisotropic AuBP used herein. Thus, the precise coating thicknesses might deviate for our samples, but the trend can reasonably be expected to be the same, considering that the Flory radii *R*_F_ exhibit a very similar trend (*R*_F_(PSSH_2k_) = 3.3 nm and *R*_F_(PSSH_12k_) = 9.5 nm, using *R*_F_ = *bN^ν^* with Kuhn length *b* = 1.8 nm for polystyrene, *N* = a number of Kuhn segments; 2.7 for PSSH_2k_ and 16.0 for PSSH_12k_ and the Flory parameter *ν* ≈0.6 for a good solvent^[^ ^27^ ^]^). The self‐assembly of CTAC‐coated AuBP, AuBP@CTAC, from aqueous dispersions can yield well‐ordered densely packed structures as described in detail in previous work.^[^ ^9^ ^]^ Although the self‐assembly conditions are not the same (self‐assembly from water, different ligand, and no liquid subphase), a tendency toward more dense packing can be expected for the PSSH_2k_ coatings with a thickness that is more comparable to CTAC‐ than PSSH_12k_‐based coatings. Indeed, we find much larger aligned domains in thin‐film supercrystals of the AuNP@PSSH_2k_ and a more pronounced correlation of AL and SL, Figure 2A,B. However, the orientation toward the substrate discussed above was random in both cases. Accordingly, the stronger correlation of the AL in the case of AuBP@PSSH_2k_ is due to their more pronounced azimuthal alignment in comparison to AuBP@PSSH_12k_. The according HR‐TEM and selected‐area electron diffraction measurements are provided in Figures S16 and S17 (Supporting Information) along with HR‐TEM measurements of the crystal structure of the AuBP in Figures S18 and S19 (Supporting Information). By varying the ligand length and thereby the thickness of the coating it is possible to change the packing density in the superstructures and simultaneously control alignment and the correlation of AL and SL.

Ding et al. very recently reported extended monolayers of AuBP and demonstrated their potential for surface‐enhanced Raman scattering (SERS).^[^ ^18b^ ^]^ Their structures differed from our superlattices, which may be due to the weakly coordinating polystyrene ligand (pentaethylenehexamine‐terminated) that was used in Ref.[^[^ ^18b^ ^]^] to facilitate the chemisorption of the SERS reporter molecules. The different binding group changes the grafting densities and conformations of the chemisorbed polymer ligands which in turn may affect the self‐assembly. In addition, an excess of ligands was used to vary the supercrystal structure in Ref.,[^[^ ^18b^ ^]^] whereas we minimized the amount of free ligand by thorough purification. Finally, ethylene glycol was used as a liquid‐subphase in their experiments, in contrast to diethylene glycol in our study, which might affect the superstructure although most likely to a lesser degree. In contrast to the monolayers reported by Ding et al. for PSSH_2.5k_ coatings, we find no overlapping of the tip‐aligned bipyramids in the 2D projection for the AuBP@PSSH_2k_ samples. This underlines the delicate role of the coating details, even when the ligand type –polystyrene‐based in both studies– is the same. The general trend of more disordered monolayers with increasing ligand length (tested up to PSSH_12k_) was also observed in the data reported by Ding et al.^[^ ^18b^ ^]^ Bi‐ or multilayers were not the focus of their study. Taken together, we can confirm less alignment in monolayers of AuBP@PSSH_12k_, but report here a structural diversity in bilayers that enabled us to experimentally address new structures and their effects on optical near‐ and far‐field as discussed in the following.

### 2.3 Optical Properties of the Superstructures

**Figure 4:**
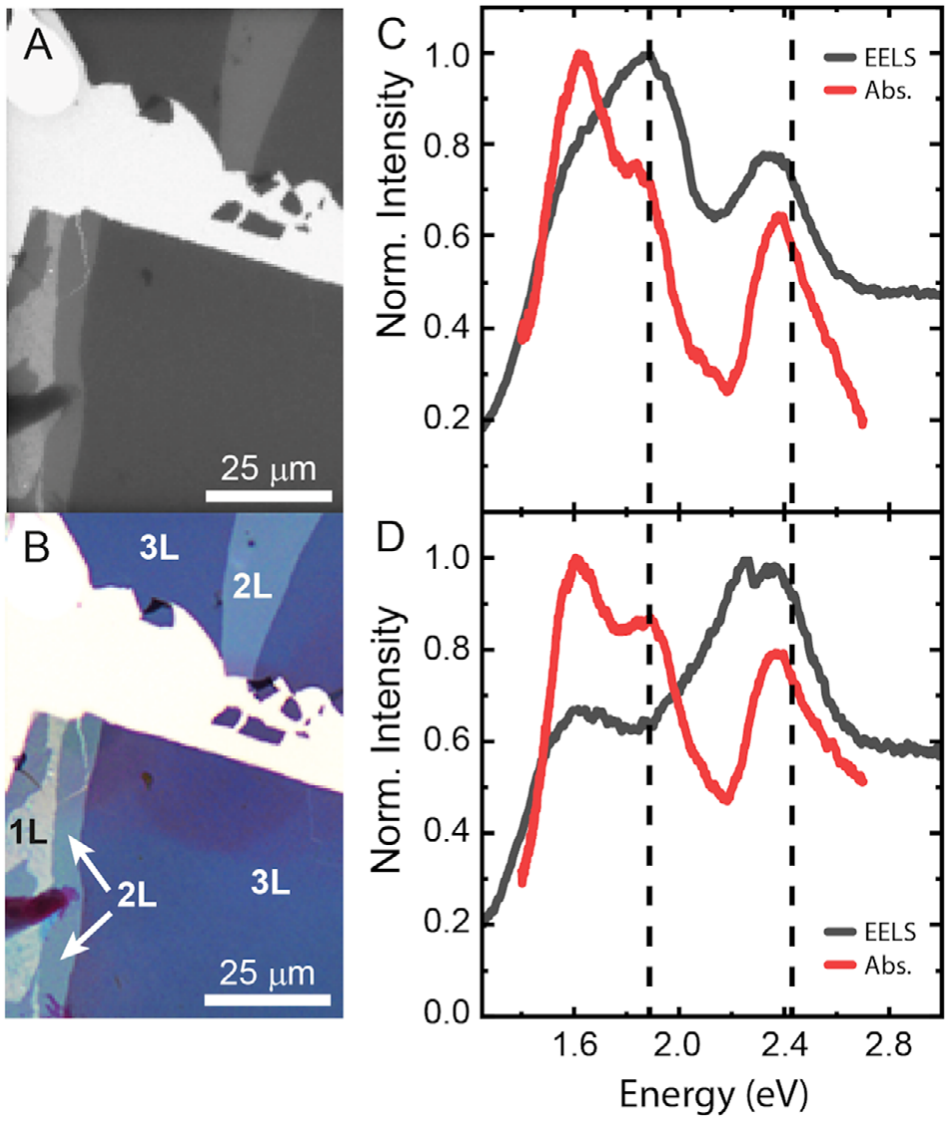
Optical microscopy and absorption of the AuBP@PSSH12k superlattices. A) Transmission electron and B) transmission optical microscopy image of an identical sample area. Absorption (red) and EELS spectra (gray) of C) a monolayer and D) a bilayer of AuBPs. The black dashed lines indicate the longitudinal and transversal mode of the AuBPs in aqueous dispersion (cf. Figure 1).

We assign the local and collective excitations of AuPB superlattices from a correlation of TEM images, EELS experiments, and micro‐absorbance spectroscopy. Far‐field absorption of well‐defined regions from AuBP@PSSH_12k_ supercrystals was measured with a micro‐absorbance setup that simultaneously records the transmission and reflection by a sample, **Figure** **4**. Similar to supercrystals of spherical AuNPs, the number of layers is determined in optical transmission microscopy based on the image contrast and color.^[^ ^2^, ^11^ ^]^ TEM images were recorded from the sample parts studied in optical spectroscopy to confirm the layer number and determine the local superlattice structure. Optical measurements were recorded on mono‐, bi‐, and trilayers, Figure 4B. The domains of the trilayers in Figure 4 cover an area of thousands of µm^2^, but the underlying structure was rather amorphous and featured only small ordered domains also in the mono‐ and bilayers as revealed by the corresponding TEM images, Figures S20 and S21 (Supporting Information). The observed variability, including the presence of amorphous regions, highlights the challenges of controlling structural order in these systems.

The absorption spectra of monolayers and bilayers, red lines in Figure 4C,D, show four overlapping peaks that arise from the individual AuBPs and the collective superlattice excitations. The peaks close to 2 and 2.5 eV are assigned to the longitudinal and transverse excitation of the individual bipyramids (dashed lines in Figure 4C,D), respectively, as identified from a comparison to the absorbance spectrum of AuBP in aqueous dispersion. The collective transverse and longitudinal modes are shifted to smaller energies compared to the individual AuBP excitations. The individual modes are clearly present in the monolayer absorption and EELS spectra (gray line) in Figure 4C, but appear only weakly in the bilayer EELS spectrum, gray line in Figure 4D, which is dominated by the collective plasmonic modes.

**Figure 5:**
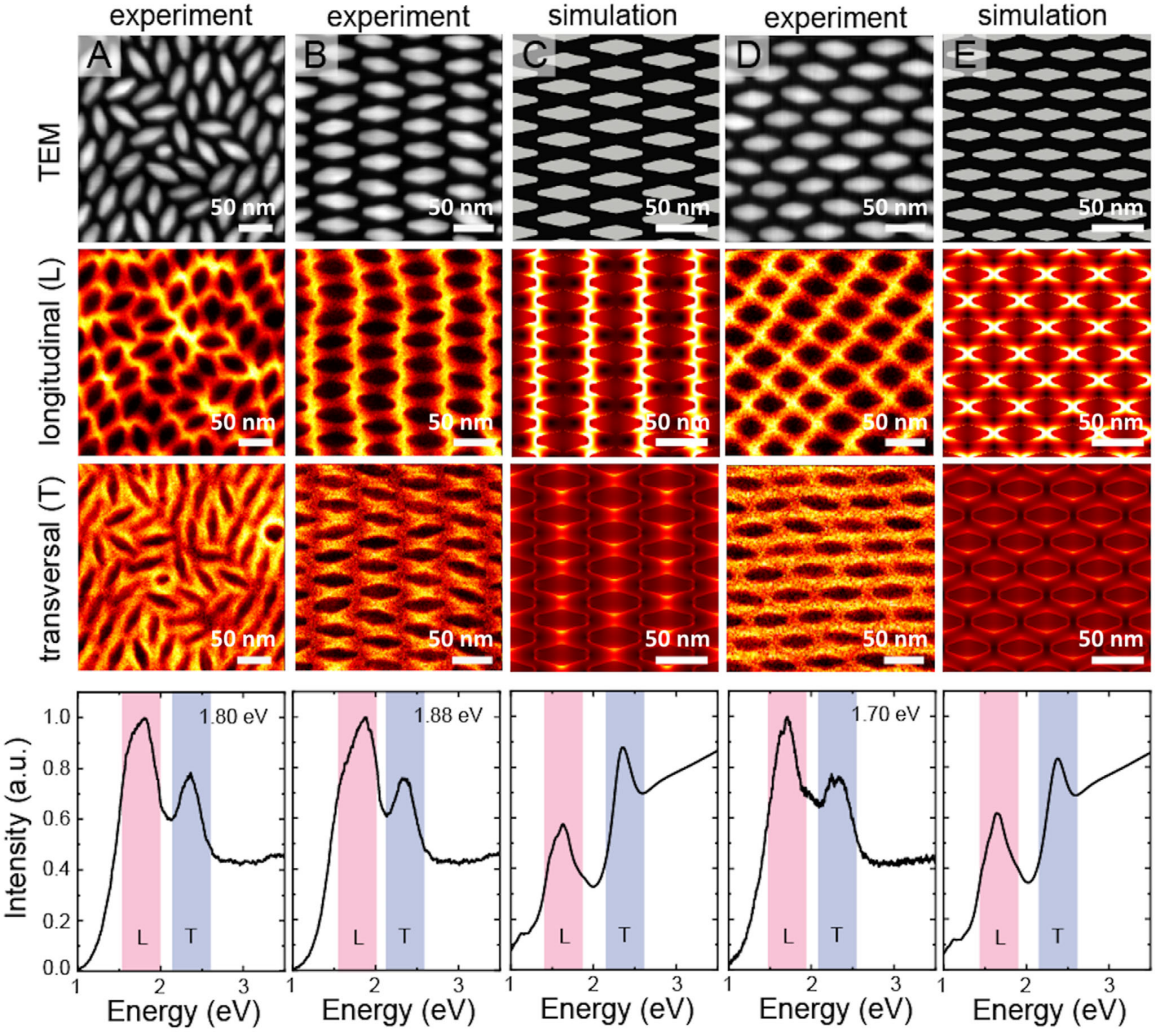
STEM‐EELS characterization and simulations of the plasmonic AuBP monolayers. A,B,D) Top: STEM‐HAADF images of thin AuBP films. Middle: The corresponding EELS filter maps of the longitudinal (L) and transversal (T) mode. Bottom: EELS spectra integrated on the corresponding whole images. The positions of the L‐ and T‐mode are indicated as red and blue bands, respectively. For each spectrum, the energy of the L‐mode is indicated. C,E) FDTD simulations of the structures in B,D). Top: Scheme of the lattice structure. Middle: The corresponding longitudinal (L) and transversal (T) near fields. Bottom: Calculated absorption spectrum for the excitation with unpolarized light. Scale bars on all images are 50 nm.

Scanning transmission electron microscopy‐electron energy loss spectroscopy (STEM‐EELS) and scanning transmission electron microscopy–high‐angle annular dark‐field (STEM‐HAADF) measurements allowed us to further correlate the microscopic structure of the AuBP superlattices with the collective plasmonic resonances, **Figure** **5**. In monolayers, distinct longitudinal and transverse plasmonic modes are identified, and the energy of the longitudinal mode of the superlattice varies with the local organization of the AuBPs. Plasmon shifts as large as 100 meV are observed in different configurations, showing that the longitudinal plasmon energy depends sensitively on local organization and superlattice symmetry. The energies of the monolayer plasmon modes, according to Figure 5A,B,D, correspond to wavelengths of ≈690, 660, and 730 nm. These strong shifts combined with the local near‐fields in the field distribution maps clearly demonstrate the character of the excitations as coupled or collective superlattice plasmon modes. Finite‐difference time‐domain (FDTD) simulations (Figure 5C,E) could reproduce the field distributions and spectra well, confirming the assignments.

**Figure 6:**
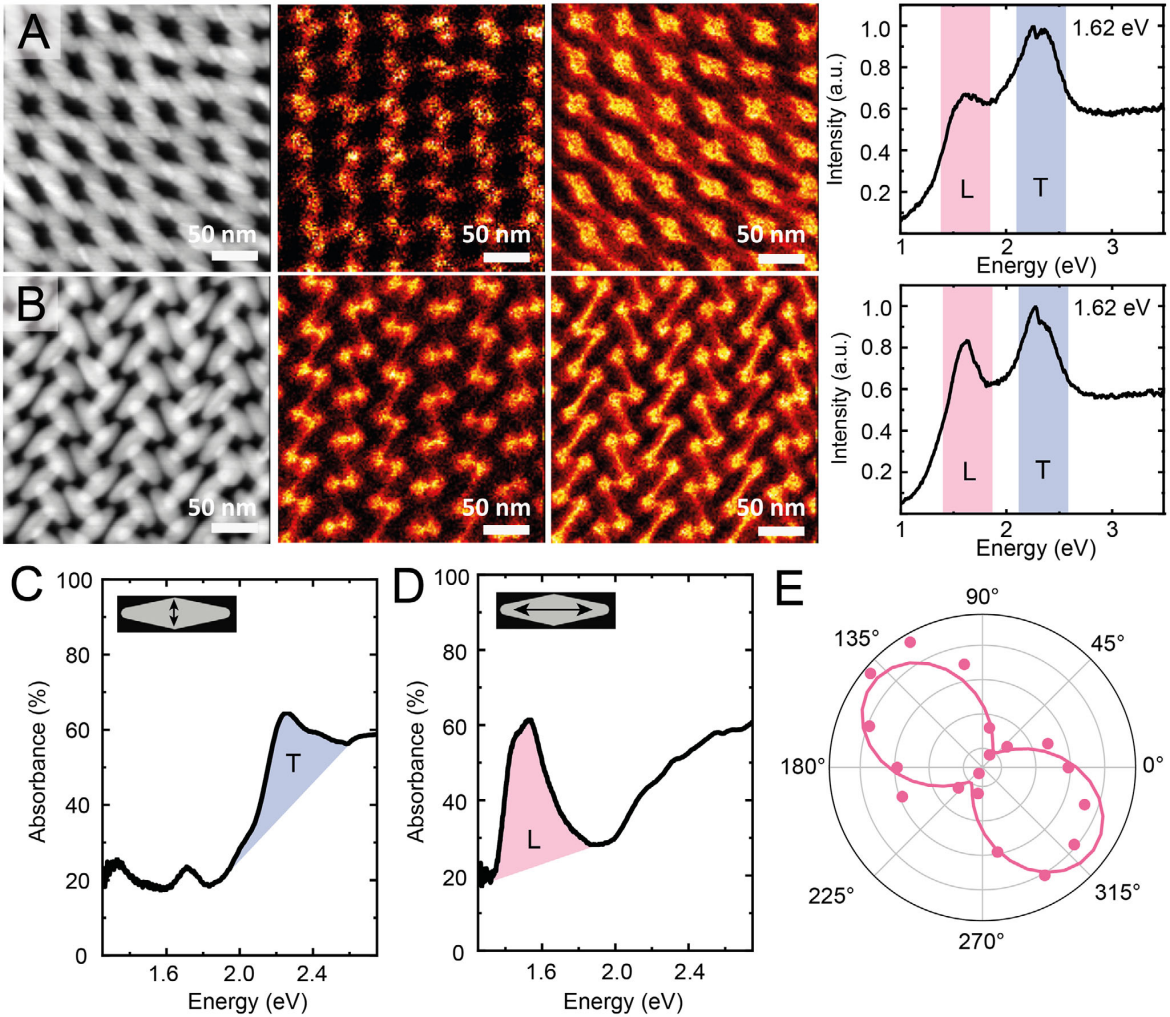
STEM‐EELS characterization and polarization‐dependence of the optical response of self‐assembled AuBP@PSSH_12k_ bilayers. A,B) Left: STEM‐HAADF images of AuBP bilayers. Middle: The corresponding EELS filter maps of the longitudinal (L) and transversal (T) mode. Right: EELS spectra integrated on the corresponding whole images. The positions of the L‐ and T‐mode are indicated as red and blue bands, respectively. For each spectrum, the energy of the L‐mode is indicated. Scale bars on all images are 50 nm. C,D) Polarization‐dependence of the absorbance of self‐assembled AuBP@PSSH_12_ films. Depending on the polarization of the light, C) the transversal (≈2.25 eV, 551 nm) or D) the longitudinal (≈1.50 eV, 827 nm) mode is excited. E) Full polarization‐dependence of the absorbance of the longitudinal mode. The spectra were recorded in an area with large crystalline bilayer domains.

While the longitudinal mode depends on the local structure, the transverse mode remains ≈2.4 eV (517 nm), similar to the energy of the mode of the AuBP in dispersion. Experimental STEM‐EELS data for bilayers are shown in **Figure** **6**. In bilayers, the longitudinal mode is shifted even more toward the infrared (1.62 eV; 765 nm) regardless of the twist angle. The relative intensity between the two modes changes in favor of the transversal mode in the bilayers as consistently observed in EELS and micro‐absorbance measurements, Figure 4 and Figure 6A,B. In addition, EELS allowed hyperspectral measurements with a spatial resolution of a few nanometers complementing the bulk measurements by studying in more detail the collective plasmonic properties of the assembled species at the single particle level. The locations of the regions of intense electric fields (hereafter termed hotspots) depend on the microscopic structure of the superlattice. The lower energy mode (longitudinal mode, L in Figures 5 and 6) corresponds to hotspots distributed at the tips of the AuBPs, whereas for the highest energy mode (transversal mode, T in Figures 5 and 6) the hotspots are localized at the base of the AuBPs. This confirms their designation as longitudinal and transverse excitations. In the randomly arranged monolayer, Figure 5A, hotspots were distributed randomly with respect to spatial location, intensity, and energy. Increasing order in the AuBP layer led to a regular spatial distribution of the near‐field hotspots with homogenous intensity and energy, Figure 5B,D. The open structures in bilayers show strongly pronounced hotspots in the voids between the assembled AuBP@PSSH (Figure 6A,B). Such voids in the superlattice should be more accessible for molecules, making this design of plasmonic metasurfaces interesting for sensing and photocatalysis applications. The EELS measurements reveal how the electric field component of light may be confined and precisely structured in self‐assembled plasmonic superlattices. The anisotropy of the plasmonic building blocks provides the possibility to switch the spatial structure of the near‐field via the energy of the light source and its polarization.

Performing polarization‐dependent optical measurements on single crystalline domains of complex superstructures is particularly challenging due to the stringent requirements for domain size, domain identification, and experimental precision. When measuring the optical spectra in the region of large crystalline bilayer domains (as shown in the Supporting Information, e.g. in Figures S4B,S8, and S9, Supporting Information) with a laser spot that is smaller than the average domain size (≈1 µm^2^ vs tens of square microns) we find a strong polarization dependence of the optical response. When the transversal mode is excited at a specific polarization angle *ϕ *(45° in Figure 6C,E), the longitudinal mode is excited for Δ*ϕ* = 90° (Figure 6D) as expected from the structural and near‐field characterization. The full polarization dependence of the longitudinal mode absorbance is shown in Figure 6E. The polarization‐dependence of the transverse mode absorbance is rotated by 90° and measuring with unpolarized light reveals both modes (Figure S22, Supporting Information). With increasing control of plasmonic supercrystal structure toward larger domain sizes, such features provide intriguing opportunities for the design of plasmonic metasurfaces. For instance, from the full optical characterization (Figure S23, Supporting Information) it can be appreciated that along with the absorbance, the reflectance and transmittance change substantially with *ϕ*, in particular in the NIR region.

## 3 Conclusion

We presented the growth of AuBP thin‐film superlattices using polystyrene ligands to stabilize the nanoparticles. The superlattices feature a great variety of microscopic structures ranging from disordered and amorphous to well‐defined 2D lattices with different packing densities and angular alignment. With HR‐TEM, a very localized correlation of atomic lattice and superlattice was observed for AuBP with a comparably thick coating (AuBP@PSSH_12k_). In contrast, a thinner coating (AuBP@PSSH_2k_) led to more densely packed and aligned lattices that lacked structural diversity in bilayers. In spherical AuNP supercrystals, in contrast, the molecular weight of the polystyrene ligands tailors the packing fraction,^[^ ^27b^ ^]^ but has no influence on crystal structure. For the anisotropic AuBP reported here, the softness of the ligands affects packing fraction as well as superlattice structures offering novel routes for further manipulation. Our analysis indicates, that domain sizes, microscopic structure, and correlation of atomic and super‐lattice are all affected by the nanoparticle ligands that, in addition, also allow some control over the bilayer twist angle and moiré structure. The combination of shape anisotropy and soft interaction provides tools to explore a larger structural diversity than is achievable with isotropic building blocks.

The symmetry and thickness of the superlattices lead to strongly structured near‐fields that can be switched/altered by exciting the structure with different energies. The near‐ and far‐field measurements demonstrate how the local field can be spatially structured with nanoscale resolution in the superlattices. Since some of the superlattices feature nanoscale voids with strong hotspots, it is conceivable to utilize such superstructures for sensing and photocatalytic experiments, even without the removal of the ligands. While the plasmonic superstructures presented here hold promise for such applications, further dedicated studies are needed to demonstrate their performance. The structural and optical insights provided in this work lay the groundwork for future investigations into these applications. For large domains, the optical response in the far‐field is polarization‐dependent and aligns well with the structural and near‐field characterization. The combination of anisotropic building blocks with distinct plasmonic properties and soft ligands therefore provides a diversity of new supercrystal structures and plasmonic properties to explore.

## 4 Experimental Section

### Materials

All chemicals were obtained from commercial suppliers and used without further purification: hexadecyltrimethylammonium bromide (CTAB, ≥ 99%), hexadecyltrimethylammonium chloride (CTAC, 25 wt. % in H_2_O), benzyldimethylhexadecylammonium chloride (BDAC), hydrogen tetrachloroaurate trihydrate (HAuCl_4_·3H_2_O, ≥ 99.9%), silver nitrate (AgNO_3_, ≥ 99.0%), l‐ascorbic acid (AA, ≥ 99%), sodium borohydride (NaBH_4_, 99%), trisodium citrate dihydrate (≥ 99.0%), toluene (≥ 99.5%), tetrahydrofuran (≥ 99.5%), ethanol (denat., > 96%) and diethylene glycol (DEG, reagent grade). Thiolated polystyrenes (PSSH, PSSH_2k_: *M*_n_ = 2000 g mol^−1^, *M*_w_ = 2300 g mol^−1^; PSSH_5k_: *M*_n_ = 5300 g mol^−1^, *M*_w_ = 5800 g mol^−1^; PSSH_12k_: *M*_n_ = 11 500 g mol^−1^, *M*_w_ = 12 400 g mol^−1^) were from Polymer Source (Canada). Ultrapure water was used for all syntheses in water.

### Gold Nanobipyramids (AuBP) Synthesis

AuBP were synthesized by a seed‐mediated growth method adapted from the literature.^[^ ^28^ ^]^ For the synthesis of the pentatwinned seeds, an aqueous CTAC solution (33 mL, 60.6 mm) was prepared in a 100 mL Erlenmeyer flask. Under gentle stirring, aqueous HAuCl_4_ (400 µL, 25 mm) and sodium citrate (4 mL, 50 mm) were added in this sequence. The reaction mixture was kept in a water bath at 30 °C for 10 min. Then, a freshly prepared NaBH_4_ solution (2.4 mL, 10 mm) was injected under vigorous stirring. A reddish solution was obtained after aging the samples for 5 days at 40 °C. This seed solution can be stored for at least one month at room temperature.

For the synthesis of AuBP, aqueous AgNO_3_ (1 mL, 10 mm), HAuCl_4_ (2 mL, 25 mm), and HCl (2 mL, 1 m) were sequentially added to a CTAB solution (100 mL, 0.1 m) under gentle stirring. Then, aqueous AA (800 µL, 100 mm) was injected under vigorous stirring, followed by the addition of 1000 µL seed solution. The reaction mixture was left undisturbed at 30 °C for 4 h. After synthesis, the nanoparticles were centrifuged to remove the excess surfactant. The bipyramid shape yield was further increased by depletion‐induced flocculation, using BDAC as a depletant.^[^ ^29^ ^]^ Supernatants were discarded and pellets were redispersed in 2.5 mm CTAC. Then, the nanoparticles were purified by at least 3 rounds of centrifugation and pellet redispersion in 2.5 mm CTAC.

### AuBP Functionalization and Self‐Assembly

The concentrated aqueous stock of AuBP was ≈500 nm (particle concentration, calculated from the Au(0) concentration using the volume of a AuBP. The Au(0) concentration was determined from the absorbance at 400 nm as described by Scarabelli et al.^[^ ^30^ ^]^). 200 µL of the stock were pipetted into a PSSH solution in THF (5 mL, 0.5 mm) and reacted overnight. Then the THF was completely removed under reduced pressure and the residue was redispersed in 1 mL toluene. The dispersion was purified by repeated centrifugation (4 centrifugations, 10 000 g, 15 min, supernatants replaced with toluene). The final pellet was brought to a volume of 1 mL with toluene. The grafting densities of the PSSH‐ligands were estimated based on thermogravimetric analysis using a thermal analyzer (STD 650, Waters, TA instruments, US) considering the geometry of the AuBP.^[^ ^9^ ^]^ A grafting density of (1.57 ± 0.25) nm^2^ was obtained for AuBP@PSSH_2k_, (0.78 ± 0.10) nm^2^ for AuBP@PSSH_5k_ and (0.26 ± 0.10) nm^−2^ for AuBP@PSSH_12k_.

For self‐assembly on the liquid subphase DEG, 100 µl AuBP@PSSH was mixed with 100 µL toluene and carefully pipetted onto the subphase (1800 µL DEG in a teflon well with 1 cm diameter and a volume of ≈2.5 mL). The well was covered with a glass cover slide to slow down the evaporation. Thin crystalline films floating on the liquid subphase had formed after ≈24 h.

### Transmission Electron Microscopy (TEM)

The TEM measurements for standard characterization were performed using a Jeol JEM‐1011 instrument operating at 100 kV. Samples of self‐assembled films were carefully skimmed off with a carbon‐coated copper grid or SiN‐grids with 3 × 3 arrays of 100 µm x 100 µm windows, (15 nm SiN membrane thickness, 200 µm frame thickness, Ted Pella, US) held by a tweezer. The grids were dried in a vacuum for at least 1 h. The samples on SiN grids were used for EELS experiments and micro‐absorbance spectroscopy. High‐resolution TEM measurements were done with a JEOL JEM‐2200FS at 200 kV.

### Electron Energy Loss Spectroscopy (EELS)

Spatially resolved EELS experiments have been performed with a NION‐Hermes 200 monochromated scanning transmission electron microscope (STEM) fitted with a Quantum Detector Merlin direct electron detector. An acceleration voltage of 60 kV, was used and a moderate monochromation led to a typical spectral resolution of 20 meV, enabling both a relatively high signal‐to‐noise ratio and enough spectral resolution to disentangle different plasmon modes. Incident and acceptance semi‐angles were 10 and 30 mrd, respectively. Hyperspectral EELS maps and high‐angle annular dark‐field measurements (HAADF) were obtained by scanning the beam and acquiring an EELS spectrum and a HAADF intensity at each scanning point. Typical scanning size and sampling were 1.5 nm and 20k points.

### Micro‐Absorbance

The absorbance was measured using a homebuilt setup. The setup consists of an inverted microscope (Olympus IX71). A broadband supercontinuum laser was used as the light source. The light entered the microscope and was focused onto the sample by a 100x objective (NA = 0.9). The transmitted light was collected by a second objective (NA = 0.9) and sent through a fiber to an Avantes spectrometer. To measure the reflected light, a beam splitter was installed in the beam path. A fiber‐coupled collimator collects the reflected light and sends it to the spectrometer. The absorption was then calculated as A(%) = 100 – R(%) – T(%) (R = Reflectance, T = Transmission). For the polarization‐dependent measurements, a polarizer and a half‐wave plate were added to the laser beam before the sample. The polar plots were obtained by plotting the area of the longitudinal/transversal mode (absorbance) over the polarization angle of the light.

### Simulations

The FDTD simulations for the bipyramid structure were configured using the commercial software package Lumerical FDTD Solutions. The bipyramids were constructed with a long (short) axis length of 50 nm (22 nm). The unit cell dimensions for the structures in Figure 5C,E were inferred from the TEM measurements and set to (100 × 33) nm and (60 × 60) nm, respectively. The mesh size was set to 0.5 nm using a mesh override region, and the electric fields were recorded in the plane normal to the incidence of light and cutting through the center of the nanoparticles. The dielectric function of gold from Olmon et al.^[^ ^31^ ^]^ was used and a background dielectric index of 1.4.

## Conflict of Interest

The authors declare no conflict of interest.

## Author Contributions

J.M. and S.J. contributed to conceptualization, formal analysis, investigation, data curation, writing, review, editing, and visualization; J.B.‐C. contributed to investigation and writing, review and editing; X.L. contributed to formal analysis, investigation, writing, review, editing, and visualization; C.G., A.K., and W.P. contributed to investigation and writing, review and editing; F.L. contributed to investigation, formal analysis, and writing, review and editing; W.J.P. contributed resources, writing, review, editing, supervision, and funding acquisition; M.K. contributed to formal analysis, investigation, resources, data curation, writing, review, editing, supervision, and project administration; M.I.‐C. contributed to conceptualization, formal analysis, investigation, writing, review, editing, and supervision; S.R. contributed to conceptualization, formal analysis, resources, data curation, writing, original draft, writing, review, editing, supervision, project administration, and funding acquisition; C.H. contributed to conceptualization, formal analysis, investigation, resources, data curation, writing, original draft, writing, review, editing, visualization, supervision, project administration, and funding acquisition; and F.S. contributed to conceptualization, formal analysis, investigation, resources, data curation, writing, original draft, writing, review, editing, visualization, supervision, project administration, and funding acquisition.

## Supporting information



Supporting Information

## Data Availability

The data that support the findings of this study are available from the corresponding author upon reasonable request.
