# Supplementary material for: Plasmonic Polymorphs by Combining Shape Anisotropy and Soft Interactions in Bipyramid Thin Films
Source: Small. 2025 May 30;21(31):2500389. doi: 10.1002/smll.202500389 (PMC12332831; doi:10.1002/smll.202500389)
Supplement: Supplementary file 1 — Supporting Information [file SMLL-21-2500389-s001.docx]

Supporting Information

**Plasmonic Polymorphs by Combining Shape Anisotropy and Soft Interactions in Bipyramid Thin Films**

Jules Marcone, Sabrina Juergensen, Juan Barrios-Capuchino, Xiaoyian Li, Claire Goldmann, Andrea Köppen, Winnie Pfeiffer, Felix Lehmkühler, Wolfgang J. Parak, Mathieu Kociak, Marianne Impéror-Clerc, Stephanie Reich, Cyrille Hamon,* Florian Schulz*


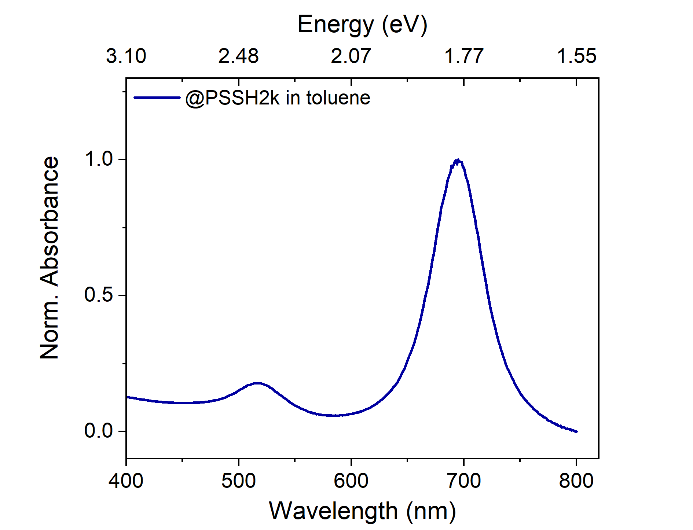


**Figure S1.** Normalized absorbance of AuBP@PSSH_2k_ in toluene.





**Figure S2.** TEM of a AuBP@PSSH_12k_ thin-film superstructure showing the transition from monolayer to bi-, tri- and multilayer (four layers and more). The ordered domains are very small and localized and the structure seems rather amorphous in many areas.





**Figure S3.** TEM of a AuBP@PSSH_12k_ thin-film superstructure showing a monolayer region with very limited and localized order.


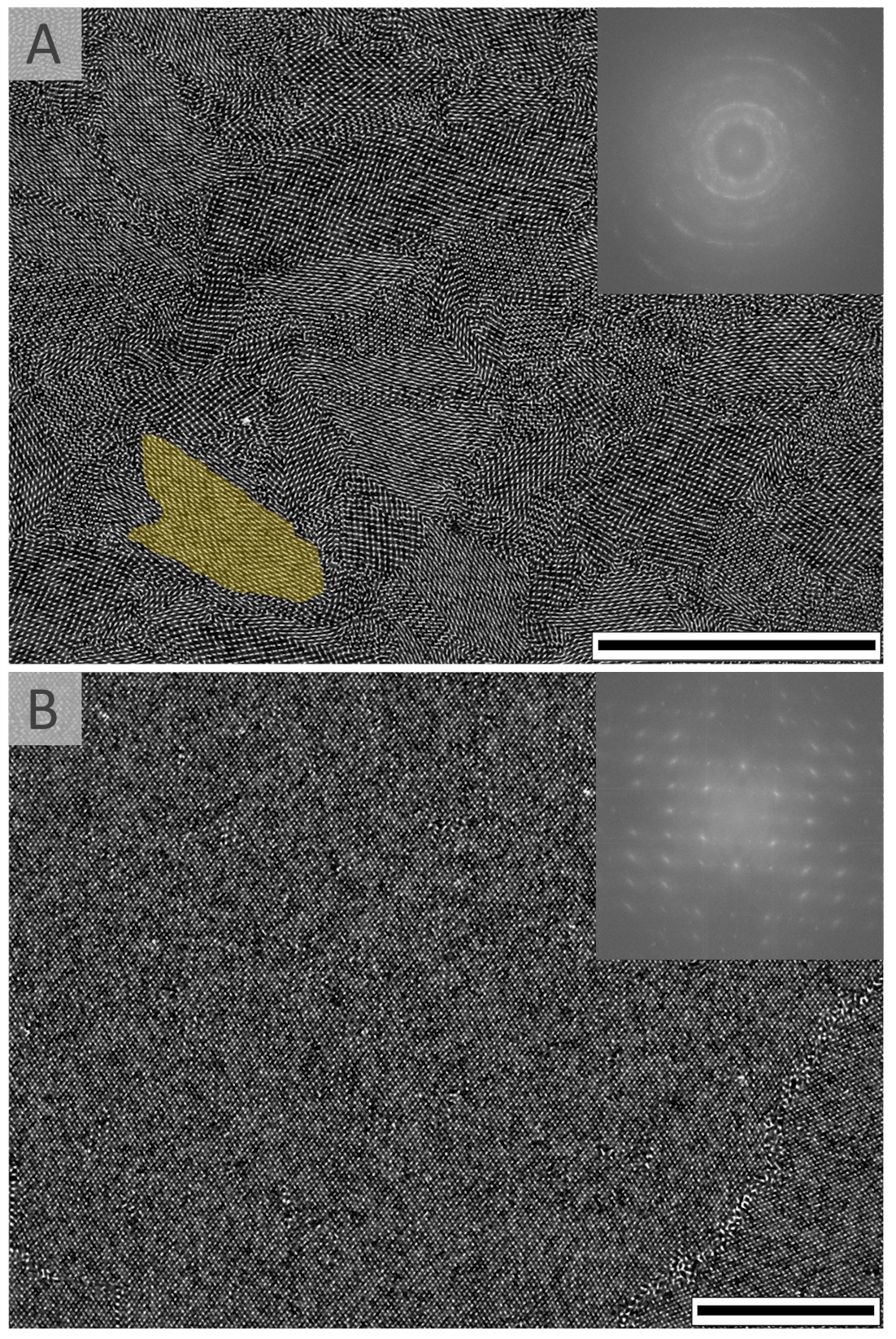


**Figure S4.** TEM of a AuBP@PSSH_12k_ thin-film superstructure showing bilayer regions with A) small (< 1 µm^2^) and B) comparably large (> 10 µm^2^) crystalline domains. Insets show the FFT of the digital images. The yellow area in A highlights a single domain. Scalebars A: 2 µm, B: 1 µm.





**Figure S5.** TEM of a AuBP@PSSH_12k_ thin-film superstructure. Densely packed bilayer structure with a twisting angle of ~90°.





**Figure S6.** TEM of a AuBP@PSSH_12k_ thin-film superstructure. Crack in a bilayer domain with a twist angle ~90°.





**Figure S7.** TEM of a AuBP@PSSH_12k_ thin-film superstructure. Bilayer area near a crack with a slightly different (more open) arrangement compared to Figure S6.





**Figure S8.** TEM of a AuBP@PSSH_12k_ thin-film superstructure. Domains with different crystal structures in a bilayer region.





**Figure S9.** TEM of a AuBP@PSSH_12k_ thin-film superstructure. Large domains in a bilayer region, where grain boundaries and stacking faults can be appreciated.





**Figure S10.** TEM of a AuBP@PSSH_12k_ thin-film superstructure. Densely packed bilayer structure with twisting angle ~90°.





**Figure S11.** TEM of a AuBP@PSSH_12k_ thin-film superstructure. Local aligned structure in a bilayer.





**Figure S12.** TEM of a AuBP@PSSH_2k_ thin-film superstructure. Despite some defects, the alignment of the AuBP with this thinner coating was ranging much longer compared to the PSSH_12k_ coating. In contrast to the localized aligned domains of a few hundred nanometers or less observed there, for this sample alignments reaching the micrometer-range were frequently observed.





**Figure S13.** TEM of a AuBP@PSSH_2k_ thin-film superstructure. Larger magnification of an aligned domain.

**

**

**Figure S14.** TEM of a AuBP@PSSH_5k_ thin-film superstructure. The ligand length (PSSH_5k_, *M*_n_ ~ 5000 g/mol) is in between PSSH_2k_ and PSSH_12k_. Neither pronounced alignment in monolayers (left) as for AuBP@PSSH_2k_ nor crystalline domains in bilayers (right) as for AuBP@PSSH_12k_ were observed.

**

**

**Figure S15.** TEM of thin-film superstructures formed by small ellipsoids (long axis ~ 21 nm,,short axis ~ 10 nm) coated with PSSH_2k_ (left column) or PSSH_12k_ (right column).





**Figure S16.** HR-TEM and SAED measurements of the assembled AuBP@PSSH_12k_ (A and B) and AuBP@PSSH_2k_ (C and D). A and C show the domains corresponding to the SAED measurements in B and D, respectively. Scalebars 100 nm (A and C) and 2 nm^-1^ (B and D).





**Figure S17.** HR-TEM and SAED measurements of the assembled AuBP@PSSH_2k_. Same area as in Figure S14C but at increasing magnifications. The increasing correlation of the atomic lattices (less angular variations of the Bragg peaks) with increasing magnification is visible. Scalebars 100 nm (A), 20 nm (C) and 2 nm^-1^ (B and D).





**Figure S18.** HR-TEM measurement of the assembled AuBP@PSSH_2k_ at large magnification. The fringes due to the interference of the diffraction of two fcc domains within each BP can be discerned.





**Figure S19.** HR-TEM measurement of the assembled AuBP@PSSH_2k_ at large magnification reveals the crystal structure of the fcc domains in the AuBP. The small crystalline object close to the center is a contamination.





**Figure S20.** TEM measurement of the monolayer-bilayer transition in the sample area shown in Figure 4 in the main text (thin-film superstructure of AuBP@PSSH_12k_). The structure was rather amorphous with comparably small ordered domains. The dots are vertically aligned AuBP.





**Figure S21.** TEM measurement of the trilayer shown in Figure 4 in the main text (thin-film superstructure of AuBP@PSSH_12k_). The structure was rather amorphous.


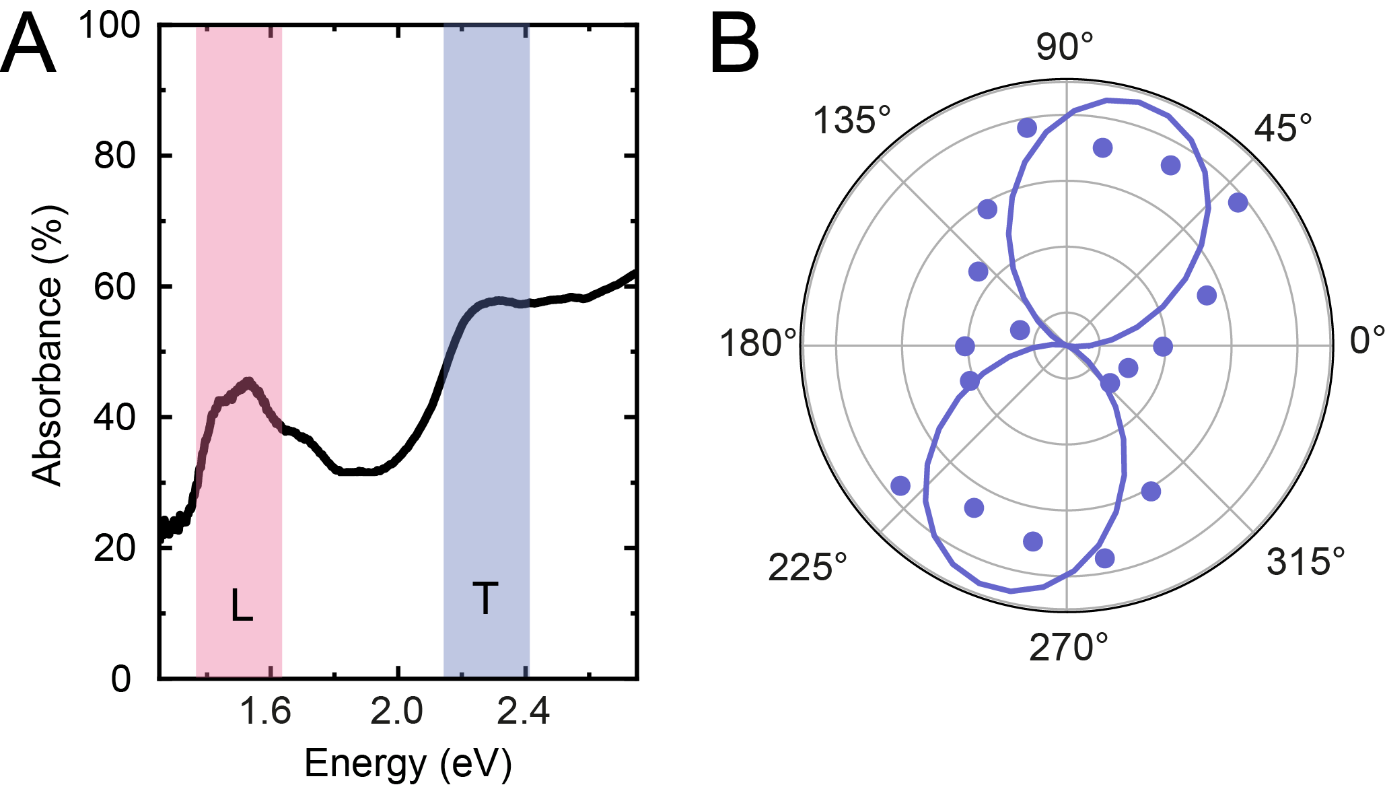


**Figure S22.** A) Absorption spectrum for the excitation with unpolarized light. Both, the L- and T-mode are visible and equally strong. B) Full polarization-dependence of the absorbance of the transversal mode in A which is rotated by 90° to the longitudinal mode. The spectra were recorded in an area with large crystalline bilayer domains. The according spectra for polarized excitation and the polarization dependence of the longitudinal mode are shown in the main text, Figure 6C-E.


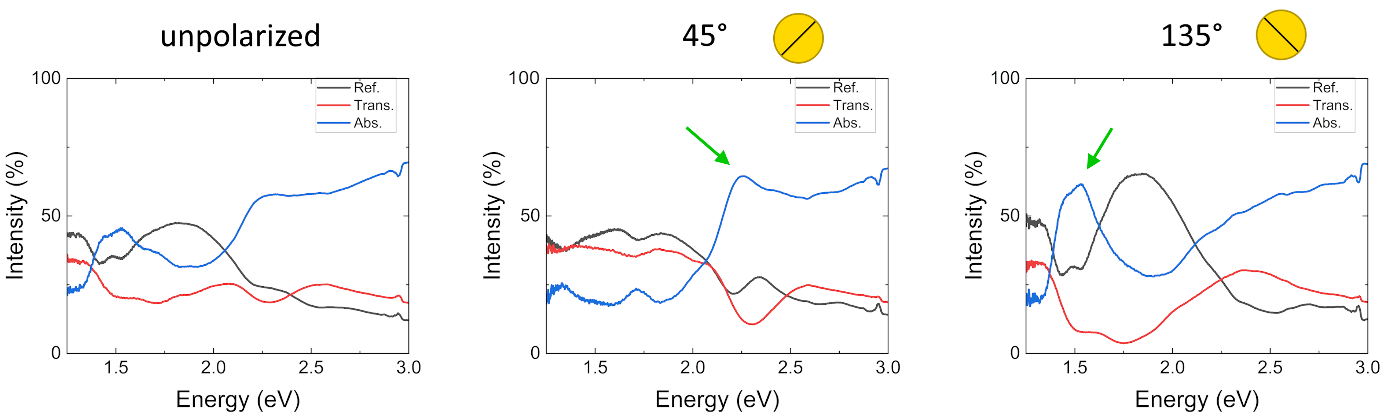


**Figure S23.** Full optical characterization (black: reflectance, red: transmission, blue: absorbance) at the same spot discussed in Figure S22 and Figure 6C-E in the main text (the absorbance spectra are the same as in Figure S22A: unpolarized, Figure 6C: 45°, Figure 6D: 135°).
